# Prognostic implications of left ventricular ejection fraction trajectory changes in heart failure

**DOI:** 10.3389/fcvm.2023.1232404

**Published:** 2023-08-23

**Authors:** Zijie Ding, Jinping Si, Xuexia Zhang, Yuze Hu, Xinxin Zhang, Yanli Zhang, Ying Liu

**Affiliations:** ^1^ Department of Cardiology, The First Affiliated Hospital of Dalian Medical University, Dalian, China; ^2^ Department of Cardiology, Shandong Health Group Zibo Hospital, Zibo, China

**Keywords:** left ventricular ejection fraction, trajectory, changes, heart failure, prognosis

## Abstract

**Aims:**

The latest guidelines recommended to assess the trajectory of left ventricular ejection fraction (LVEF) in patients with heart failure (HF). However, there is limited data on the trajectory of LVEF in real-world settings. In this study, we investigated the frequency and prognostic implications of changes in LVEF trajectory.

**Methods:**

Patients were divided into intensified LVEF, static LVEF, and worsening LVEF groups based on the transitions of HF types from baseline to follow-up. The intensified and worsening LVEF groups were further subdivided into mild (≤10% absolute changes of LVEF) and significant (>10% absolute changes of LVEF) increase or decrease groups according to the magnitude of change. The incidences and associations of changes in LVEF with patient outcomes were analyzed.

**Results:**

Among the 2,429 patients in the study cohort, 38.3% of HF with reduced ejection fraction (HFrEF) and 37.6% of HF with mildly reduced ejection fraction (HFmrEF) showed an improvement in their LVEF. In contrast, a decline in LVEF was observed in 19.3% of HF patients with preserved ejection fraction (HFpEF) and 34.9% of those with HFmrEF. Cox regression analysis showed that the intensified LVEF group was associated with a lower risk of composite endpoints, while the worsening LVEF group yielded opposite findings. Subgroup analysis revealed that compared to those with mild changes in LVEF, baseline HFrEF patients with significant increase showed a lower risk of composite outcome, while baseline HFpEF patients were the opposite.

**Conclusions:**

The trajectories of LVEF changes are strongly correlated with outcomes in patients with HF who had prior history of HF admission. The most significant prognostic implications observed in patients with significant LVEF changes. Trajectory LVEF and type of HF changes are useful tools recommended for prognostication.

## Introduction

Heart failure (HF) is a complex clinical syndrome characterized by symptoms and signs that result from impaired ventricular filling or ejection of blood caused by structural or functional abnormalities. Echocardiographic measurement of left ventricular ejection fraction (LVEF) is essential in classifying patients with heart failure, given its ability to predict their response to therapies and overall prognosis (1). HF can be divided into 3 categories based on baseline LVEF: HF with reduced ejection fraction (HFrEF) (LVEF ≤40%), HF with mildly reduced ejection fraction (HFmrEF) (LVEF 41%–49%), and HF with preserved ejection fraction (HFpEF) (LVEF ≥50%) (2–6). While this stratification approach can assist physicians in terms of diagnosis and management, it is worth noting that HF is a heterogeneous syndrome, and LVEF may fluctuate over time (7–9). According to the 2022 American Heart Association (AHA)/American College of Cardiology (ACC)/Heart Failure Society of America (HFSA) guidelines for the management of HF, patients with HF are typically in a dynamic trajectory, emphasizing the initial classification of HF based on LVEF and reclassification in accordance with the serial assessment (5). To date, the dynamic alterations in LVEF trajectory across the entire HF spectrum have been largely underexplored. Furthermore, whether outcomes differ among patients experiencing diverse LVEF changes remains unclear. The primary objective of the current study was to investigate the occurrence of LVEF trajectory changes in real-world patients with HF and their associated outcomes. Additionally, our study aimed to assess the prognostic implications of varying changes in LVEF levels.

## Materials and methods

This was a retrospective, single-center, observational, real-world study approved by the institutional review board of Dalian Medical University. All the procedures were conducted in accordance with the principles of the Declaration of Helsinki and its subsequent amendments.

### Study population, clinical definitions, and classification

**Figure 1:**
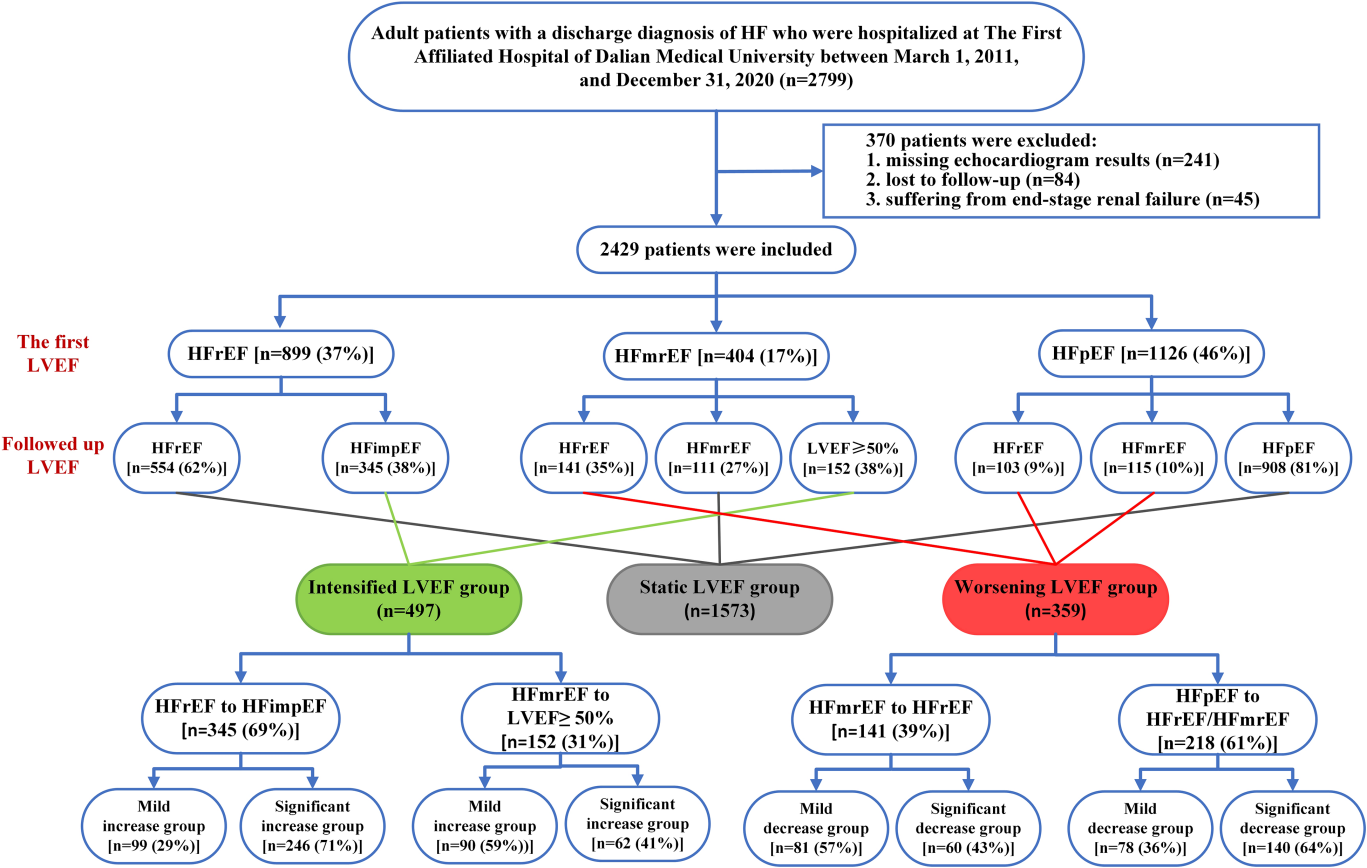
Flowchart of the study. HF, heart failure; LVEF, left ventricular ejection fraction; HFrEF, HF with reduced ejection fraction; HFmrEF, HF with mildly reduced ejection fraction; HFpEF, HF with preserved ejection fraction.

We retrospectively collected a cohort of inpatients at The First Affiliated Hospital of Dalian Medical University between March 1, 2011 and December 31, 2020. Patients were included if they: (1) were aged >18 years; (2) had history of HF hospitalization and discharge diagnosis of HF (ICD 10 code: I50.900); (3) had twice or more echocardiogram data with an interval at least 6 months apart. We used Yidu Cloud to seek participants for the enrolment process with the terms mentioned above. When the patient underwent more than two echocardiograms, the first and last measurements during the follow-up were considered to assess the change in LVEF and twice echocardiography data were collected during hospitalization. We excluded patients who met any of the following criteria: (1) missing echocardiogram results, (2) lost to follow-up, or (3) had end-stage renal failure. HF was diagnosed according to the 2022 AHA/ACC/HFSA HF guidelines and defined as: (i) HFrEF if initial LVEF was ≤40%; (ii) HFmrEF if initial LVEF was 41%–49%; (iii) HFpEF if initial LVEF was ≥50%; (iv) HFimpEF if initial LVEF ≤40% and LVEF >40% at follow-up; additional criteria for HFmrEF and HFpEF comprised objective evidence of spontaneous or provokable raised left ventricular (LV) filling pressures including increased natriuretic peptide, invasive/noninvasive hemodynamic measurement suggesting elevated LV filling pressures. The study participants were divided into three groups based on the changes in HF status. Group 1, the intensified LVEF group, consisted of patients who transitioned from HFrEF to HFimpEF (HFrEF to HFmrEF/LVEF ≥50%) and HFmrEF to LVEF ≥50%. Group 2, the worsening LVEF group comprised patients shifting from HFpEF to HFmrEF, HFpEF to HFrEF, and HFmrEF to HFrEF. Finally, group 3, the static LVEF group consisted of patients with absense of any changes in their HF types. Subgroup analyses were carried out to investigate the potential prognostic implications of varying degrees of LVEF changes. The intensified LVEF group was stratified into two subgroups based on the magnitude of their LVEF increase: mild increase (0%–10% absolute increase of LVEF) and significant increase (>10% absolute increase of LVEF). Likewise, patients in the worsening LVEF group were categorized into two subgroups based on the degree of their LVEF decrease: mild decrease (0%–10% absolute decrease of LVEF) and significant decrease (>10% absolute decrease of LVEF). The flowchart of our study is shown in Figure 1.

### Clinical data

Baseline demographics, laboratory data, comorbidities, and pharmaco- therapeutics were recorded by trained specialists. LVEF were measured by standard 2D transthoracic echocardiographic examination. The preferred method for assessing LVEF was the quantitative 2D biplane volumetric Simpson method from the 4- and 2-chamber views. Other echocardiographic parameters, including left atrial volume index (LAVI), left ventricular end-diastolic dimension (LVEDD), right ventricular diameter (RVD) were also collected using the American Society of Echocardiography Guidelines by 2 to 3 sonographers (10).

### Clinical outcome and follow up

The primary endpoint was a composite outcome including all-cause mortality or HF-related admissions. Transitions between the HF types were evaluated based on measurements of LVEF at baseline and follow-up. All enrolled patients were required to return to the outpatient clinic for follow-up on a regular basis. In cases where patients missed their scheduled appointments, annual phone interviews were conducted. The study endpoint was determined as either June 30th, 2021 or the incidence of HF-related admissions or death.

### Statistical analysis

Statistical analysis was performed with Statistical Package for Social Sciences, version 25 (SPSS Inc., Chicago, IL, USA). Qualitative variables were summarized as numbers (proportions), and the *χ*^2^ test or Fisher's exact test was used to compare groups based on the researchers’ discretion. For quantitative variables, data with normal distribution were expressed as means ± standard deviations, and ANOVA was applied to between-group comparison, while data with non-normal distribution were expressed as the median (interquartile range), and the Kruskal–Wallis test was used for multi-group comparisons. Kaplan–Meier analysis was performed to demonstrate the cumulative incidence of adverse events, and the log-rank test was conducted to compare differences. All variables reported in Table 1 were included in the univariate Cox regression analysis. Subsequently, multivariate Cox regression models were applied to evaluate the associations among the trajectory (intensified, static, or worsening) and extent (mild or significant) of changes in LVEF and adverse outcome, including covariates with a *P*-value <0.05 in univariate analyses or acknowledged as vital factors in previous studies. The hazard ratio (HR) and their corresponding 95% confidence intervals (95% CI) were calculated. A 2-sided *P*-value <0.05 was statistically significant.

**Table 1:** Baseline characteristics of the overall population.

	Entire population (*n* = 2,429)	Intensified LVEF group (*n* = 497)	Static LVEF group (*n* = 1,573)	Worsening LVEF group (*n* = 359)	*P* value
Age, years	68 (61, 75)	65 (57, 72)^a,c^	69 (62, 75)	68 (61, 76)	<0.001
Male gender, *n*	1,482 (61.0%)	342 (68.8%)^a^	905 (57.5%)	235 (65.5%)^b^	<0.001
Heart rate, b.p.m.	78 (66, 94)	84 (71, 103)^a,c^	77 (65,93)	78 (66,93)	<0.001
Diastolic BP, mm Hg	81 ± 14	84 ± 16^a,c^	80 ± 13	80 ± 12	<0.001
Systolic BP, mm Hg	139 ± 24	137 ± 24	139 ± 24	140 ± 23	0.270
Medical history					
CAD, *n*	1,116 (45.9%)	238 (47.9%)	684 (43.5%)	194 (54.0%)^b^	0.001
Prior MI, *n*	535 (22.0%)	84 (16.9%)ac	358 (22.8%)	93 (25.9%)	0.004
Valvular heart disease, *n*	398 (16.3%)	54 (10.9%)^a,c^	288 (18.3%)	56 (15.6%)	<0.001
Hypertension, *n*	1,665 (68.5%)	316 (63.6%)^a,c^	1,089 (69.2%)	260 (72.4%)	0.014
Diabetes, *n*	948 (39.0%)	188 (37.8%)	605 (38.5%)	155 (43.2%)	0.211
Atrial flutter, *n*	93 (3.8%)	28 (5.6%)^a^	49 (3.1%)	16 (4.5%)	0.031
Paroxysmal AF, *n*	177 (7.2%)	29 (5.8%)	121 (7.7%)	27 (7.5%)	0.375
Persistent AF, *n*	648 (26.6%)	133 (26.8%)	433 (27.5%)	82 (22.8%)	0.194
Pharmaco-therapeutics					
Diuretic, *n*	1,572 (64.7%)	350 (70.4%)^a,c^	1,020 (64.8%)	202 (56.3%)^b^	<0.001
Nitrate, *n*	880 (36.2%)	151 (30.4%)^a,c^	576 (36.6%)	153 (42.6%)^b^	0.001
Digoxin, *n*	285 (11.7%)	70 (14.1%)	180 (11.4%)	35 (9.7%)	0.126
Beta-blocker, *n*	1,796 (73.9%)	417 (83.9%)^a,c^	1,117 (71.0%)	262 (73.0%)	<0.001
ACEI or ARB or ARNI, *n*	1,359 (55.9%)	316 (63.6%)^a,c^	864 (54.9%)	179 (49.9%)	<0.001
Spirolactone, *n*	1,421 (58.5%)	347 (69.8%)^a,c^	901 (57.3%)	173 (48.2%)^b^	<0.001
SGLT2i, *n*	25 (1.0%)	13 (2.6%)^a,c^	11 (0.7%)	1 (0.3%)^b^	<0.001
NOAC, *n*	285 (11.7%)	90 (18.1%)^a,c^	170 (10.8%)	25 (7.0%)^b^	<0.001
Warfarin, *n*	456 (18.7%)	66 (13.3%)^a,c^	323 (20.5%)	67 (18.7%)	0.001
Antiplatelet, *n*	1,416 (58.2%)	293 (59.0%)	906 (57.6%)	217 (60.4%)	0.581
Lipid-lowering drug, *n*	1,652 (68.0%)	341 (68.6%)	1,075 (68.3%)	236 (65.7%)	0.602
Device therapy					
ICD, *n*	21 (0.9%)	5 (1.0%)	12 (0.8%)	4 (1.1%)	0.648
CRT or CRTD, *n*	18 (0.7%)	5 (1.0%)	12 (0.8%)	1 (0.3%)	0.468
Laboratory values					
Hemoglobin, g/L	136.3 ± 20.4	139.6 ± 20.6^a,c^	135.5 ± 19.9	135.0 ± 22.0	<0.001
BNP level, pg/ml	327.3 (118.6, 783.6)	421.1 (151.7, 1,014.9)^a^	305.1 (106.6, 728.2)	333.4 (152.0, 741.4)	<0.001
Creatinine, mmol/L	76.0 (61.0, 94.0)	77.0 (62.0, 93.0)	75.0 (60.0, 92.5)	80.0 (64.0, 100.0)^b^	0.003
Uric acid, mmol/L	374.0 (305.0, 454.8)	388.0 (310.5, 479.0)^a^	370.0 (303.0, 443.0)	378.0 (305.0, 462.0)	0.023
Echocardiographic parameters					
LAVI, ml/m^2^	32.2 (25.4, 42.7)	33.5 (27.8, 41.9)	31.7 (24.5, 42.8)	32.2 (25.7, 42.7)	0.170
LVEDD, mm	53.0 (48.0, 59.0)	55.0 (50.6, 60.0)^a,c^	52.0 (47.0, 59.0)	52.0 (48.0, 57.0)	<0.001
RVD, mm	18.0 (17.0, 20.0)	18.0 (16.5, 20.0)	18.0 (17.0, 20.0)	18.0 (17.0, 20.0)	0.144
LVEF, %	47.0 (36.0, 57.0)	38.0 (33.0, 43.0)^a,c^	55.0 (36.0, 58.0)	50.0 (45.0, 56.0)^b^	<0.001
E/e’	11.6 (9.0, 15.0)	11.4 (9.0, 14.5)	11.5 (9.0, 15.4)	12.0 (9.2, 16.5)	0.087
Time interval between two echocardiograms, years	2.0 (1.0, 3.4)	1.7 (1.0, 2.9)^a,c^	2.1 (1.0, 3.5)	2.3 (1.2, 3.8)	<0.001

BP, blood pressure; CAD, coronary artery disease; MI, myocardial infarction; AF, atrial fibrillation; ACEI, angiotensin-converting enzyme inhibitor; ARB, angiotensin II receptor blocker; ARNI, angiotensin receptor–neprilysin inhibitor; NOAC, novel oral anticoagulants; ICD, implantable cardioverter defibrillator; CRT, cardiac resynchronization therapy; CRTD, cardiac resynchronization therapy defibrillator; BNP, B-type natriuretic peptide; LAVI, left atrial volume index; LVEDD, left ventricular end-diastolic dimension; RVD, right ventricular diameter; LVEF, left ventricular ejection fraction.

Values are mean ± SD, median (IQR), or *n* (%).

^a^ *P *< 0.05 between intensified LVEF group and static LVEF group.

^b^ *P *< 0.05 between worsening LVEF group and static LVEF group.

^c^ *P *< 0.05 between intensified LVEF group and worsening LVEF group.

## Results

### Patient population

**Figure 2:**
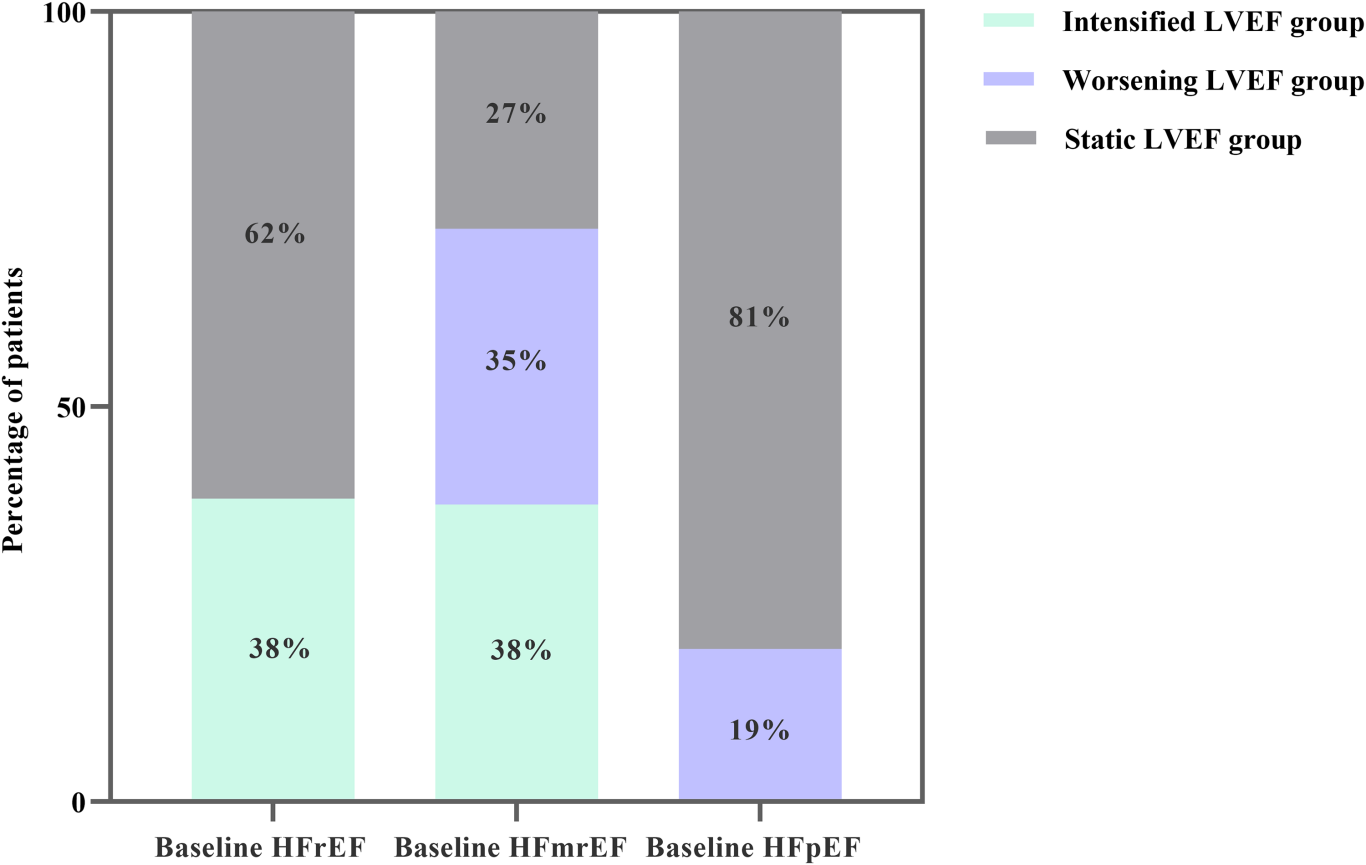
Column chart for the frequency of LVEF trajectory changes in real-world patients with heart failure categorized by the baseline types of heart failure.

A total of 2,799 patients were initially included in our study. After excluding patients missing echocardiogram results (*n* = 241), lost to follow-up (*n* = 84), and suffering from end-stage renal failure (*n* = 45), 2,429 were finally enrolled. At baseline, most cases presented HFpEF (46%), followed by HFrEF (37%) and HFmrEF (17%). At follow-up, 497 (20%) patients showed an intensified LVEF, 1,573 (65%) patients reported static LVEF, and 359 (15%) patients experienced worsening LVEF (Figure 1). Based on the data presented in Figure 2, 62% (*n* = 554) of baseline HFrEF patients remained in the same category, while 38% (*n* = 345) were classified as intensified LVEF group. Among the baseline HFmrEF patients, 38% (*n* = 152) experienced intensified LVEF, while 35% (*n* = 141) regressed to worsening LVEF group. The majority of baseline HFpEF patients (81%, *n* = 908) remained static LVEF, with 19% (*n* = 218) dropping to worsening LVEF group.

### Baseline characteristics

The baseline characteristics of different groups are summarized in Table 1. The group of patients with an intensified LVEF was characterized by a younger age, more often male, and higher diastolic blood pressure and heart rate than the static and worsening LVEF groups. Regarding medical history, prior myocardial infarction, hypertension, and valvular heart disease were less common in the intensified LVEF group than in the other two groups. The prevalence of coronary artery disease and atrial flutter were comparable between the intensified and worsening LVEF groups but higher than in the static LVEF group. In terms of medication, patients in the intensified LVEF group were prescribed diuretics, ACE inhibitors, angiotensin receptor blockers, or angiotensin receptor-neprilysin inhibitors (ACEI/ARB/ARNI), β-blockers, spironolactone, sodium-glucose co-transporter inhibitors (SGLT2i) and non vitamin K oral anticoagulants more often than those in the static and worsening LVEF groups, while warfarin therapy use was less frequent. An analysis of laboratory test results and echocardiographic findings revealed that the intensified LVEF group had higher hemoglobin levels and a greater LVEDD but a lower LVEF than the other two groups. BNP levels were highest in the intensified LVEF group and lowest in the static LVEF group.

### Adverse cardiovascular outcomes

**Figure 3:**
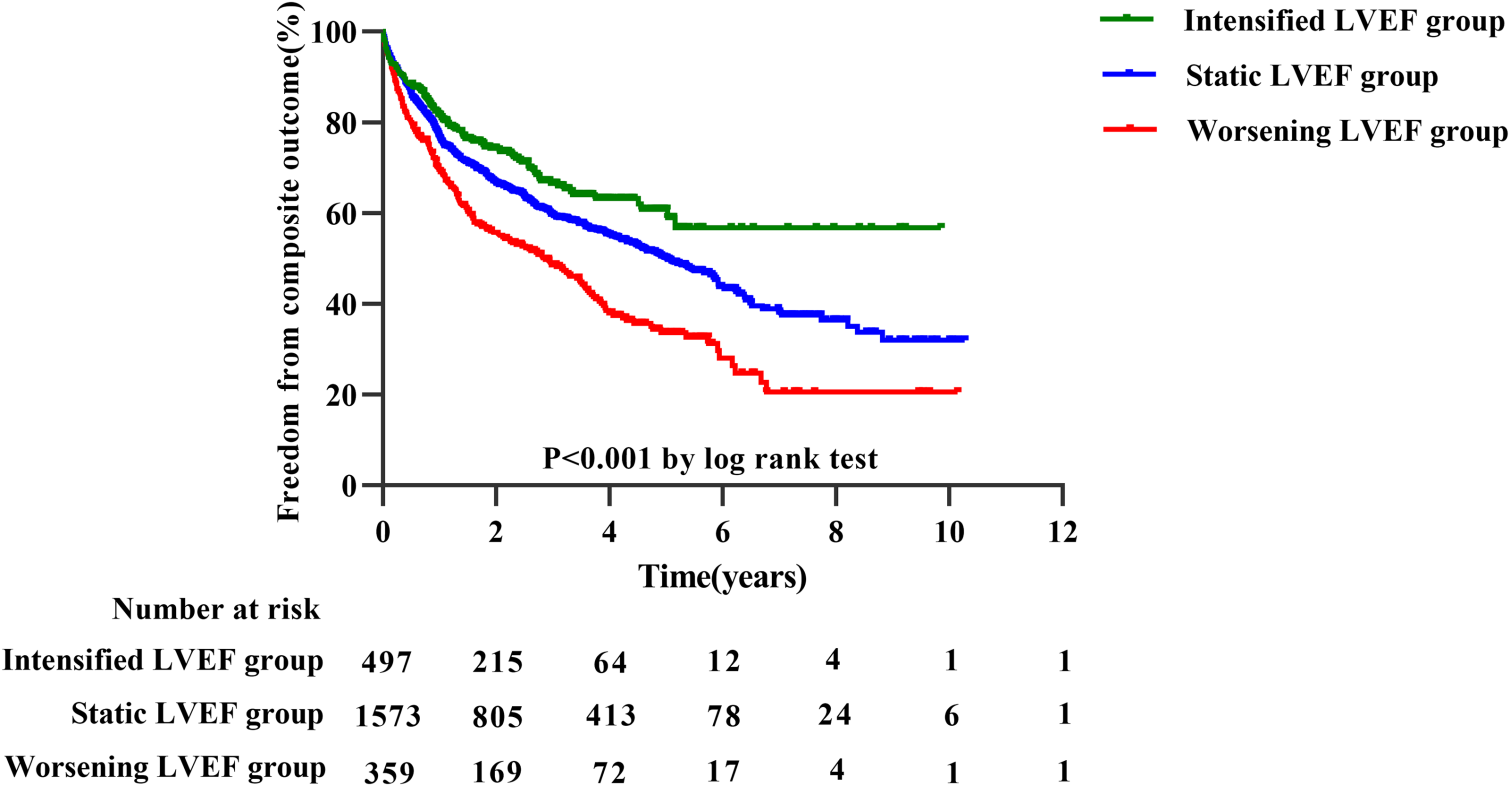
Kaplan–Meier estimates for composite outcomes in three groups divided by trajectory changes in LVEF.

The median follow-up was 3.78 years, during which 1,029 experienced a terminal event, including 86 deaths [intensified/static/worsening LVEF: 11 (2%)/54 (3%)/21 (6%)], 943 HF-related admissions [intensified/static/worsening LVEF: 132 (27%)/620 (39%)/191 (53%)]. Patients with intensified LVEF showed a lowest cumulative incidence of composite outcome (log-rank *P *< 0.001) (Figure 3). Furthermore, after adjusting for various potential confounders, Cox regression analysis demonstrated that compared with static LVEF, intensified LVEF was associated with significant reduced risk of the composite end points [adjusted HR (aHR) 0.621 [95% CI, 0.514–0.751], *P *< 0.001], whereas worsening LVEF was associated with an increase in risk [aHR 1.698 (95% CI, 1.446–1.994), *P *< 0.001] (Table 2), The relationships between changes of LVEF trajectory and HF-related admissions or all-cause mortality were shown in Supplementary Table S1.

**Table 2:** Cox regression to evaluate the associations between changes of LVEF trajectory and composite outcome.

	Unadjusted			Adjusted^a^		
	HR	95% CI	*P* value	HR	95% CI	*P* value
Composite outcome						
Intensified LVEF group	0.763	0.636–0.914	0.003	0.621	0.514–0.751	<0.001
Static LVEF group	1	Reference	NA	1	Reference	NA
Worsening LVEF group	1.486	1.274–1.734	<0.001	1.698	1.446–1.994	<0.001

^a^ Multivariable Cox regression model adjusted for age, sex, baseline heart rates, baseline systolic blood pressure, history of prior MI, history of valvular heart disease, history of hypertension, history of diabete, history of atrial flutter, use of ACEI or ARB or ARNI, use of beta-blocker, use of spirolactone, use of SGLT2i, creatinine, baseline LVEF, and time interval between two echocardiograms. LVEF, left ventricular ejection fraction; HR, hazard ratio; CI, confidence interval; MI, myocardial infarction; ACEI, angiotensin-converting enzyme inhibitor; ARB, angiotensin II receptor blocker; ARNI, angiotensin receptor–neprilysin inhibitor, SGLT2i, sodium-glucose co-transporter inhibitors.

### Subgroup analyses

#### Patients with intensified LVEF

**Figure 4:**
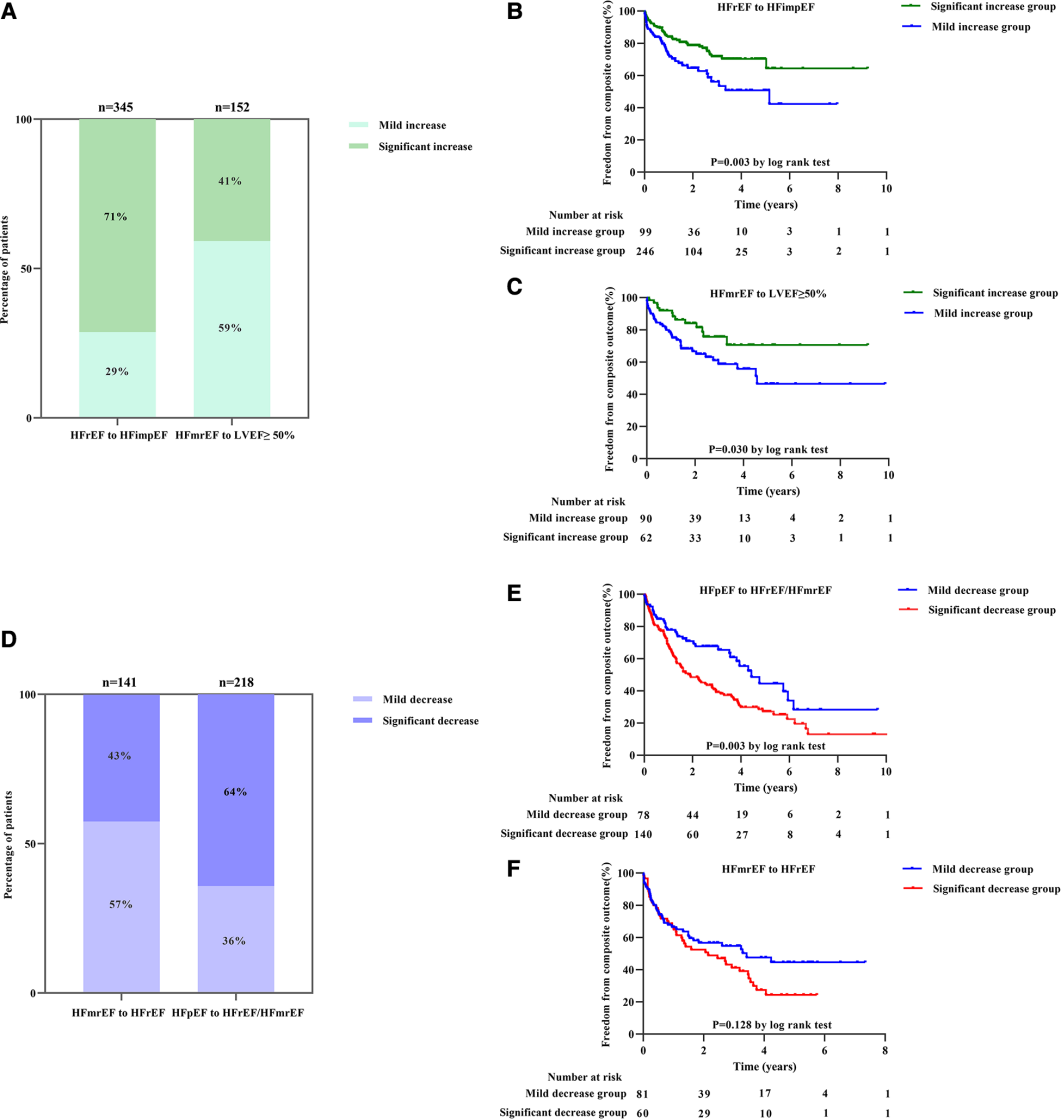
The frequency of magnitude of LVEF changes and its association with composite outcomes in intensified and worsening LVEF subgroup: (**A**) column chart for the frequency of magnitude of LVEF changes in intensified LVEF subgroup; (**B,C**) Kaplan–Meier estimates for composite outcomes in patients with intensified LVEF subgroup divided by magnitude of LVEF changes into HFrEF to HFimpEF (**B**) and HFmrEF to LVEF ≥50% (**C**) patients, respectively. (**D**) Column chart for the frequency of magnitude of LVEF changes in worsening LVEF subgroup; (**E,F**) Kaplan–Meier estimates for composite outcomes in patients with worsening LVEF subgroup divided by magnitude of LVEF changes into HFpEF to HFrEF/HFmrEF (**E**) and HFmrEF to HFrEF (**F**) patients, respectively.

The study included 497 participants who exhibited intensified LVEF at follow-up [HFrEF to HFimpEF: *n* = 345 (69%); HFmrEF to LVEF ≥50%: *n* = 152 (31%)] (Figure 1). In patients transformed from HFrEF to HFimpEF [Significant increase: *n* = 246 (71%) vs. Mild increase: *n* = 99 (29%)] (Figure 4A), 95 patients met the composite end point [Significant increase: *n* = 56 (23%) vs. Mild increase: *n* = 39 (39%)], of which 7 died [Significant increase: *n* = 6 (2%) vs. Mild increase: *n* = 1 (1%)], and 88 were rehospitalized for worsening HF [Significant increase: *n* = 50 (20%) vs. Mild increase: *n* = 38 (38%)]. Unadjusted event rates for the composite outcome in significant increase group were lower than those in mild increase group (log-rank *P = *0.003) (Figure 4B). Besides, Cox regression analysis indicated that significant increase in LVEF was associated with lower risks of composite outcome [aHR, 0.511 (95% CI, 0.314–0.832); *P = *0.007] (Table 3). Meanwhile, we found analogous results in patients transformed from HFmrEF to LVEF ≥50% (log-rank *P = *0.030) (Figure 4C), but did not show significance after adjustment for multiple confounders [aHR, 0.595 (95% CI, 0.284–1.246); *P = *0.169] (Table 3). The associations between the magnitude of LVEF increase and HF-related admissions or all-cause mortality were shown in Supplementary Table S2.

**Table 3:** Cox regression to evaluate the associations between the magnitude of LVEF increase and composite outcome in intensified LVEF group.

	Unadjusted			Adjusted^a^		
	HR	95% CI	*P* value	HR	95% CI	*P* value
HFrEF to HFimpEF						
Mild increase group	1	Reference	NA	1	Reference	NA
Significant increase group	0.547	0.363–0.823	0.004	0.511	0.314–0.832	0.007
HFmrEF to LVEF ≥50%						
Mild increase group	1	reference	NA	1	reference	NA
Significant increase group	0.500	0.264–0.945	0.033	0.595	0.284–1.246	0.169

^a^ Multivariable Cox regression model adjusted for age, sex, baseline heart rates, baseline systolic blood pressure, history of prior MI, history of valvular heart disease, history of hypertension, history of diabete, history of atrial flutter, use of ACEI or ARB or ARNI, use of beta-blocker, use of spirolactone, use of SGLT2i, creatinine, baseline LVEF, and time interval between two echocardiograms. LVEF, left ventricular ejection fraction; HR, hazard ratio; CI, confidence interval; MI, myocardial infarction; ACEI, angiotensin-converting enzyme inhibitor; ARB, angiotensin II receptor blocker; ARNI, angiotensin receptor–neprilysin inhibitor, SGLT2i, sodium-glucose co-transporter inhibitors.

#### Patients with worsening LVEF

As to the worsening LVEF group, a total of 359 patients [HFmrEF to HFrEF: *n* = 141 (39%); HFpEF to HFmrEF/HFrEF: *n* = 218 (61%)] were found in LVEF deterioration trajectory (Figure 1). In baseline HFpEF [Significant decrease: *n* = 140 (64%) vs. Mild decrease: *n* = 78 (36%)] (Figure 4D), 132 patients suffered from the composite outcome [Significant decrease: *n* = 97 (69%) vs. Mild decrease: *n* = 35 (45%)], of which 18 died [Significant decrease: *n* = 12 (9%) vs. Mild decrease: *n* = 6 (8%)], and 114 were rehospitalized for worsening HF [Significant decrease: *n* = 85 (61%) vs. Mild decrease: *n* = 29 (37%)]. The significant decrease group showed a remarkably higher cumulative incidence of composite outcome compared with the mild decrease group (log-rank *P = *0.003) (Figure 4E). In addition, Cox regression analysis revealed a higher risk of composite outcome in patients with a significant decrease in LVEF [aHR, 2.479 (95% CI, 1.590–3.867); *P < *0.001] (Table 4). In baseline HFmrEF, the event rates between the two groups were comparable (log-rank *P = *0.128) (Figure 4F), however, the differences in risk appeared in Cox regression models after adjustment [aHR, 1.717 (95% CI, 1.071–2.752); *P = *0.025] (Table 4). The associations between the magnitude of LVEF decrease and HF-related admissions or all-cause mortality were shown in Supplementary Table S3.

**Table 4:** Cox regression to evaluate the associations between the magnitude of LVEF decrease and composite outcome in worsening LVEF group.

	Unadjusted			Adjusted^a^		
	HR	95% CI	*P* value	HR	95% CI	*P* value
HFpEF to HFrEF/HFmrEF						
Mild increase group	1	Reference	NA	1	Reference	NA
Significant increase group	1.788	1.214–2.633	0.003	2.479	1.590–3.867	<0.001
HFmrEF to HFrEF						
Mild increase group	1	Reference	NA	1	Reference	NA
Significant increase group	1.403	0.905–2.176	0.130	1.717	1.071–2.752	0.025

^a^ Multivariable Cox regression model adjusted for age, sex, baseline heart rates, baseline systolic blood pressure, history of prior MI, history of valvular heart disease, history of hypertension, history of diabete, history of atrial flutter, use of ACEI or ARB or ARNI, use of beta-blocker, use of spirolactone, use of SGLT2i, creatinine, baseline LVEF, and time interval between two echocardiograms. LVEF, left ventricular ejection fraction; HR, hazard ratio; CI, confidence interval; MI, myocardial infarction; ACEI, angiotensin-converting enzyme inhibitor; ARB, angiotensin II receptor blocker; ARNI, angiotensin receptor–neprilysin inhibitor, SGLT2i, sodium-glucose co-transporter inhibitors.

## Discussion

In the present study, we demonstrated that changes in LVEF were prevalent in HF patients during follow-up. Besides, the trajectory and magnitude of LVEF changes were independently associated with patient outcomes.

Given that LVEF is a continuously varying parameter that is impacted by the characteristics and severity of HF over a period of time, relying on a solitary cutoff at a particular time point is inherently inadequate (11). According to the latest guidelines from AHA/ACC/HFSA, patients with HF typically follow a dynamic trajectory, thus the classification for baseline and subsequent LVEF was crucial (5). Moreover, patients may experience transitions between HF types with minimal or great LVEF changes (4). As such, conducting in-depth analyses of the extent of LVEF changes in different LVEF trajectories is imperative.

Our study assessed changes in LVEF trajectory by analyzing the first and last echocardiogram measurements during follow-up, resulting in 856 patients (35.2%) experiencing a shift between typical HF classification categories, consistent with the findings of Savarese et al. (12). 38.38% (*n* = 345) of patients with baseline HFrEF transitioned to HFimpEF, while 61.62% remained in the HFrEF group, similar to results observed in Farré et al. (13) and Lupó et al.'s (14) studies. Notably, patients with HFmrEF at baseline exhibited the most significant LVEF trajectory variation, with 37.62% experiencing an increase in LVEF >50% and 34.90% declining to HFrEF. The latter was broadly in line with that in the study by Savarese, et al. (12). However, Farré et al. reported that 24% of baseline HFmrEF patients had reduced LVEF at 1-year follow-up, which was lower than our study (13). Lupón et al. observed that HFmrEF patients could be distributed across HFrEF (25%), HFmrEF (39%), and HFpEF (36%) groups at the end of a 15-year follow-up (14). This discrepancy might be explained by different baseline characteristics of study populations and intervals between the two echocardiograms. A significant proportion of patients (19.36%) with HFpEF at baseline fell to LVEF <50%. This proportion was higher than reported by Lupón et al. (14). (11.1%) and Tsuji et al. (15). (10% at 1 year and 12% at 3 years), suggesting progressive systolic dysfunction in HFpEF.

Several recent clinical trials have found that LVEF changes were related to adverse outcomes (7, 16, 17). In the present study, we identified the difference in outcomes among various directions of changes in LVEF. The result implied that a change in LVEF trajectory was inversely associated with the risk of adverse outcomes. On the one hand, intensified LVEF portended a significantly better prognosis, indicating a more benign HF phenotype with spontaneous myocyte function recovery or recovery induced by guideline-directed medical and device therapies. In present study, the propotions of HF foundation medications use [ACEI/ARB/ARNI (63.6%), spironolactone (69.8%), β-blockers (83.9%) and SGLT2i (2.6%)] in intensified LVEF group were significantly higher than those in the static and worsening LVEF groups. SGLT2i were firstly recommended for treatment of HFrEF in the 2021 HF guidelines and had a recommendation (Class 2a) in HFmrEF and HFpEF in the 2022 HF guidelines. However, the dealine of enrolment was December 31, 2020 in our study, which leading to rare use of SGLT2i. On the other hand, worsening LVEF resulted in worse outcomes, possibly reflecting a more vulnerable phase in the course of HF and a progressive systolic dysfunction in HFpEF or HFmrEF.

At present, the data supporting the prognostic value of varying extent of change in LVEF is much more sparse. Most studies have used arbitrary cutoffs based on absolute values, such as a criterion of 0%–20%, to reflect changes in LV function (18–20). Several studies have established that serial LVEF change ≥10% is associated with mortality. Consequently, in the present study, we chose 10% absolute changes as a cutoff for LVEF quantification to identify patients with intensified or worsening LVEF. Regarding the improvement in LVEF, our findings were in line with a study conducted on patients with myocardial infarction (21). It was found that the survival rate of individuals who experienced a ≥10% absolute increase in LVEF was twice as high as those who did not show such an improvement after a 5-year follow-up period. Strange et al. reported that patients with a >10% absolute decline in LVEF experienced a two to eight-fold increased risk of cardiovascular-related mortality according to baseline LVEF levels (22). The present study further explored the prognostic implications of varying extent of changes in LVEF based on the shift in HF types. We observed that baseline HFrEF patients who experienced a significant increase in LVEF had a lower risk of the composite outcome. On the other hand, baseline HFpEF patients who showed a significant decrease in LVEF had a higher risk of the composite outcome. However, in baseline HFmrEF patients, mild and significant changes in LVEF showed no difference in prognosis. The results might be explained by the small sample size of baseline HFmrEF patients in our population. It requires more definitive study in a larger population. Based on our findings, a comprehensive assessment of the trajectory and degree of LVEF changes is of great necessity for predicting outcomes in patients with HF. Besides, there were still some participants who had significant changes of LVEF either increase or decrease with static HF type. A recent research confirmed that in patients with a baseline LVEF <50%, an absolute increase of 6%–12% was associated with a reduced risk of death (22). On the contrary, in terms of those with HFpEF, a significant decrease in LVEF levels below a threshold of 50%–55% were related to an increased risk of mortality (22). Therefore, baseline HFrEF patients with significant increase in LVEF may have a better outcome regardless of HF type transitions, however, for baseline HFpEF patients without the transitions, prognosis may show no relationship with LVEF decrease.

The study has several limitations. Firstly, it was a retrospective cohort study conducted at a single center with a relatively small sample size of patients. Furthermore, the timing of LVEF assessments was based on clinical decisions rather than a predefined protocol, which represents a potential source of bias. Besides, chronic HF patients without history of HF hospitalization as well as pre-HF (formerly named stage BHF) were not included. In addition, by this study classification, the prognostic outcomes in the static group with significant changed of LVEF were overlooked. In addition, the data of global longitudinal strain analysis of the left ventricle, cardiac magnetic resonance and RV function parameters were not included, which required further studies.

In conclusion, the trajectories and magnitude of LVEF changes are strongly correlated with outcomes in patients with HF who had prior history of HF admission. The prognostic implications were most apparent in the patients who experienced significant changes either increase or decrease of trajectory ejection fractions. Close relationship between significant increase in intensified LVEF trajectories and better outcome suggests that the measures which significantly improve LVEF or prevent its deterioration should be firmly endorsed. Overall, evaluating the changes in type of HF and trajectory LVEF levels can offer valuable clinical information and potentially guide clinical decision making.

## Data Availability

The raw data supporting the conclusions of this article will be made available by the authors, without undue reservation.

## References

[1] Lund LH Pitt B Metra M. Left ventricular ejection fraction as the primary heart failure phenotyping parameter. Eur J Heart Fail. (2022) 24:1158–61. 10.1002/ejhf.2576 35703027

[2] Bilchick KC Stafford P Laja O Elumogo C Bediako P Tolbert N Relationship of ejection fraction and natriuretic peptide trajectories in heart failure with baseline reduced and mid-range ejection fraction. Am Heart J. (2022) 243:1–10. 10.1016/j.ahj.2021.08.015 34453882 PMC8633031

[3] Triposkiadis F Butler J Abboud FM Armstrong PW Adamopoulos S Atherton JJ The continuous heart failure spectrum: moving beyond an ejection fraction classification. Eur Heart J. (2019) 40:2155–63. 10.1093/eurheartj/ehz158 30957868 PMC7963129

[4] Miller RJH Nabipoor M Youngson E Kotrri G Fine NM Howlett JG Heart failure with mildly reduced ejection fraction: retrospective study of ejection fraction trajectory risk. ESC Heart Failure. (2022) 9:1564–73. 10.1002/ehf2.13869 35261203 PMC9065872

[5] Heidenreich PA Bozkurt B Aguilar D Allen LA Byun JJ Colvin MM 2022 AHA/ACC/HFSA guideline for the management of heart failure. J Am Coll Cardiol. (2022) 79:e263–421. 10.1016/j.jacc.2021.12.012 35379503

[6] Gevaert AB Tibebu S Mamas MA Ravindra NG Lee SF Ahmad T Clinical phenogroups are more effective than left ventricular ejection fraction categories in stratifying heart failure outcomes. ESC Heart Failure. (2021) 8:2741–54. 10.1002/ehf2.13344 33934542 PMC8318507

[7] Ghimire A Fine N Ezekowitz JA Howlett J Youngson E McAlister FA. Frequency, predictors, and prognosis of ejection fraction improvement in heart failure: an echocardiogram-based registry study. Eur Heart J. (2019) 40:2110–7. 10.1093/eurheartj/ehz233 31280320

[8] Islam S. Transition of left ventricular ejection fraction in heart failure. Adv Exp Med Biol. (2018) 1067:5–15. 10.1007/5584_2018_178 29516307

[9] Lupon J Gavidia-Bovadilla G Ferrer E de Antonio M Perera-Lluna A Lopez-Ayerbe J Dynamic trajectories of left ventricular ejection fraction in heart failure. J Am Coll Cardiol. (2018) 72:591–601. 10.1016/j.jacc.2018.05.042 30071987

[10] Rychik J Ayres N Cuneo B Gotteiner N Hornberger L Spevak PJ American society of echocardiography guidelines and standards for performance of the fetal echocardiogram. J Am Soc Echocardiogr. (2004) 17:803–10. 10.1016/j.echo.2004.04.011 15220910

[11] Lüscher TF. The spectrum of heart failure: value of left ventricular ejection fraction and its moving trajectories. Eur Heart J. (2019) 40:2097–100. 10.1093/eurheartj/ehz454 33215630

[12] Savarese G Vedin O D'Amario D Uijl A Dahlström U Rosano G Prevalence and prognostic implications of longitudinal ejection fraction change in heart failure. JACC Heart Failure. (2019) 7:306–17. 10.1016/j.jchf.2018.11.019 30852236

[13] Farré N Lupon J Roig E Gonzalez-Costello J Vila J Perez S Clinical characteristics, one-year change in ejection fraction and long-term outcomes in patients with heart failure with mid-range ejection fraction: a multicentre prospective observational study in catalonia (Spain). Bmj Open. (2017) 7:e18719. 10.1136/bmjopen-2017-018719 PMC5778274 29273666

[14] Lupón J Gavidia-Bovadilla G Ferrer E de Antonio M Perera-Lluna A López-Ayerbe J Heart failure with preserved ejection fraction infrequently evolves toward a reduced phenotype in long-term survivors. Circ Heart Fail. (2019) 12:e005652. 10.1161/CIRCHEARTFAILURE.118.005652 30827137

[15] Tsuji K Sakata Y Nochioka K Miura M Yamauchi T Onose T Characterization of heart failure patients with mid-range left ventricular ejection fraction-a report from the CHART-2 study. Eur J Heart Fail. (2017) 19:1258–69. 10.1002/ejhf.807 28370829

[16] Wybraniec MT Orszulak M Męcka K Mizia-Stec K. Heart failure with improved ejection fraction: insight into the variable nature of left ventricular systolic function. Int J Env Res Pub He. (2022) 19:14400. 10.3390/ijerph192114400 PMC9656122 36361280

[17] Zhang Y Guallar E Blasco-Colmenares E Butcher B Norgard S Nauffal V Changes in follow-up left ventricular ejection fraction associated with outcomes in primary prevention implantable cardioverter-defibrillator and cardiac resynchronization therapy device recipients. J Am Coll Cardiol. (2015) 66:524–31. 10.1016/j.jacc.2015.05.057 26227190 PMC4522701

[18] Tayal U Prasad SK. Myocardial remodelling and recovery in dilated cardiomyopathy. JRSM Cardiovasc Dis. (2017) 6:818769017. 10.1177/2048004017734476 PMC5637962 29051817

[19] Tehrani D Seto AH. Ejection fraction as the key to improvement in ischemic cardiomyopathy outcomes. Circ Cardiovasc Interv. (2022) 15:e012000. 10.1161/CIRCINTERVENTIONS.122.012000 35411777

[20] Wilcox JE Fang JC Margulies KB Mann DL. Heart failure with recovered left ventricular ejection fraction. J Am Coll Cardiol. (2020) 76:719–34. 10.1016/j.jacc.2020.05.075 32762907

[21] Parodi G Memisha G Carrabba N Signorini U Migliorini A Cerisano G Prevalence, predictors, time course, and long-term clinical implications of left ventricular functional recovery after mechanical reperfusion for acute myocardial infarction. Am J Cardiol. (2007) 100:1718–22. 10.1016/j.amjcard.2007.07.022 18082514

[22] Strange G Playford D Scalia GM Celermajer DS Prior D Codde J Change in ejection fraction and long-term mortality in adults referred for echocardiography. Eur J Heart Fail. (2021) 23:555–63. 10.1002/ejhf.2161 33768605

