# Supplementary material for: Prognostic implications of left ventricular ejection fraction trajectory changes in heart failure
Source: Front Cardiovasc Med. 2023 Aug 23;10:1232404. doi: 10.3389/fcvm.2023.1232404 (PMC10481864; doi:10.3389/fcvm.2023.1232404)
Supplement: Supplementary file 1 [file Datasheet1.doc]

**Table S1.** Cox regression to evaluate the associations between changes of LVEF trajectory and HF-related admissions or all-cause mortality

|  | Unadjusted | | | Adjusted^a^ | | |
| --- | --- | --- | --- | --- | --- | --- |
|  | HR | 95%CI | P value | HR | 95%CI | P value |
| **HF-related admissions** |  |  |  |  |  |  |
| Intensified LVEF group | 0.761 | 0.631-0.919 | 0.005 | 0.614 | 0.504-0.748 | <0.001 |
| Static LVEF group | 1 | reference | NA | 1 | reference | NA |
| Worsened LVEF group | 1.416 | 1.204-1.666 | <0.001 | 1.576 | 1.332-1.864 | <0.001 |
| **All-cause mortality** |  |  |  |  |  |  |
| Intensified LVEF group | 0.595 | 0.391-0.906 | 0.016 | 0.571 | 0.371-0.881 | 0.011 |
| Static LVEF group | 1 | reference | NA | 1 | reference | NA |
| Worsened LVEF group | 1.561 | 1.172-2.079 | 0.002 | 1.726 | 1.274-2.337 | <0.001 |

a Multivariable Cox regression model adjusted for age, sex, baseline heart rates, baseline systolic blood pressure, history of prior MI, history of valvular heart disease, history of hypertension, history of diabete, history of atrial flutter, use of ACEI or ARB or ARNI, use of beta-blocker, use of spirolactone, use of SGLT2i, creatinine, baseline LVEF, and time interval between two echocardiograms. LVEF, left ventricular ejection fraction; HR, hazard ratio; CI, confidence interval; MI, myocardial infarction; ACEI, angiotensin-converting enzyme inhibitor; ARB, angiotensin II receptor blocker; ARNI, angiotensin receptor–neprilysin inhibitor, SGLT2i, sodium-glucose co-transporter inhibitors.

**Table S2.** Cox regression to evaluate the associations between the magnitude of LVEF increase and HF-related admissions or all-cause mortality in intensified LVEF group

|  | Unadjusted | | | Adjusted^a^ | | |
| --- | --- | --- | --- | --- | --- | --- |
|  | HR | 95%CI | P value | HR | 95%CI | P value |
| **HF-related admissions** |  |  |  |  |  |  |
| **HFrEF to HFimpEF** |  |  |  |  |  |  |
| Mild increase group | 1 | reference | NA | 1 | reference | NA |
| Significant increase group | 0.497 | 0.326-0.758 | 0.001 | 0.484 | 0.293-0.800 | 0.005 |
| **HFmrEF to LVEF≥50%** |  |  |  |  |  |  |
| Mild increase group | 1 | reference | NA | 1 | reference | NA |
| Significant increase group | 0.506 | 0.260-0.982 | 0.044 | 0.545 | 0.251-1.187 | 0.126 |
| **All-cause mortality** |  |  |  |  |  |  |
| **HFrEF to HFimpEF** |  |  |  |  |  |  |
| Mild increase group | 1 | reference | NA | 1 | reference | NA |
| Significant increase group | 0.649 | 0.246-1.711 | 0.382 | 0.636 | 0.202-2.000 | 0.438 |
| **HFmrEF to LVEF≥50%** |  |  |  |  |  |  |
| Mild increase group | 1 | reference | NA | 1 | reference | NA |
| Significant increase group | 0.491 | 0.099-2.434 | 0.384 | 1.150 | 0.076-17.492 | 0.920 |

a Multivariable Cox regression model adjusted for age, sex, baseline heart rates, baseline systolic blood pressure, history of prior MI, history of valvular heart disease, history of hypertension, history of diabete, history of atrial flutter, use of ACEI or ARB or ARNI, use of beta-blocker, use of spirolactone, use of SGLT2i, creatinine, baseline LVEF, and time interval between two echocardiograms. LVEF, left ventricular ejection fraction; HR, hazard ratio; CI, confidence interval; MI, myocardial infarction; ACEI, angiotensin-converting enzyme inhibitor; ARB, angiotensin II receptor blocker; ARNI, angiotensin receptor–neprilysin inhibitor, SGLT2i, sodium-glucose co-transporter inhibitors.

**Table S3.** Cox regression to evaluate the associations between the magnitude of LVEF decrease and HF-related admissions or all-cause mortality in worsening LVEF group

|  | Unadjusted | | | Adjusted^a^ | | |
| --- | --- | --- | --- | --- | --- | --- |
|  | HR | 95%CI | P value | HR | 95%CI | P value |
| **HF-related admissions** |  |  |  |  |  |  |
| **HFpEF to HFrEF/HFmrEF** |  |  |  |  |  |  |
| Mild decrease group | 1 | reference | NA | 1 | reference | NA |
| Significant decrease group | 1.848 | 1.212-2.818 | 0.004 | 2.364 | 1.475-3.789 | <0.001 |
| **HFmrEF to HFrEF** |  |  |  |  |  |  |
| Mild decrease group | 1 | reference | NA | 1 | reference | NA |
| Significant decrease group | 1.470 | 0.939-2.299 | 0.092 | 1.806 | 1.110-2.938 | 0.017 |
| **All-cause mortality** |  |  |  |  |  |  |
| **HFpEF to HFrEF/HFmrEF** |  |  |  |  |  |  |
| Mild decrease group | 1 | reference | NA | 1 | reference | NA |
| Significant decrease group | 1.921 | 0.980-3.766 | 0.057 | 2.314 | 1.048-5.109 | 0.038 |
| **HFmrEF to HFrEF** |  |  |  |  |  |  |
| Mild decrease group | 1 | reference | NA | 1 | reference | NA |
| Significant decrease group | 0.687 | 0.249-1.897 | 0.469 | 1.154 | 0.328-4.063 | 0.824 |

a Multivariable Cox regression model adjusted for age, sex, baseline heart rates, baseline systolic blood pressure, history of prior MI, history of valvular heart disease, history of hypertension, history of diabete, history of atrial flutter, use of ACEI or ARB or ARNI, use of beta-blocker, use of spirolactone, use of SGLT2i, creatinine, baseline LVEF, and time interval between two echocardiograms. LVEF, left ventricular ejection fraction; HR, hazard ratio; CI, confidence interval; MI, myocardial infarction; ACEI, angiotensin-converting enzyme inhibitor; ARB, angiotensin II receptor blocker; ARNI, angiotensin receptor–neprilysin inhibitor, SGLT2i, sodium-glucose co-transporter inhibitors.
